# Joint Trajectories of Spousal Social Support and Depressive Symptoms in Older Age

**DOI:** 10.1177/0898264317747077

**Published:** 2017-12-14

**Authors:** Mai Stafford, Toni C. Antonucci, Paola Zaninotto

**Affiliations:** 1 University College London, UK; 2 University of Michigan, Ann Arbor, USA

**Keywords:** intimate partner, personal relationships, latent growth curve

## Abstract

**Objective:** We describe changes in depressive symptoms and positive and negative social support from the spouse/partner in a representative sample of older people in England. **Method:** Men and women aged 50+ (*N* = 7,171) from the English Longitudinal Study of Ageing reported social support and depressive symptoms (Center for Epidemiologic Studies Depression Scale) on up to five occasions between 2002-2003 and 2010-2011. Parallel process latent growth models estimated their bidirectional associations, adjusted for gender, wealth, education, and limiting illness. **Results:** In age- and gender-adjusted models, positive spousal support decreased and negative support increased over time, especially among women. Greater increases over time in depressive symptoms were seen in those with lower positive support or higher negative support at baseline. More baseline depressive symptoms predicted greater declines in positive support and greater increases in negative support from the spouse. **Discussion:** Improving older couple’s relationship quality may help reduce depressive symptoms.

## Introduction

Many studies show an association between social support and lower risk of depressive symptoms (Schwarzbach, Luppa, Forstmeier, König, & Riedel-Heller, 2014). Social support may have a direct protective effect or may buffer the effect of stressors on depressive symptoms. In addition, this association may be bidirectional, such that psychological disorder leads to poor quality social support (Monroe, 1983). Older adults have been the focus of much of the research in this field for at least two reasons. First, they are thought to be at greater risk of deficits in their social relationships, and second, they may benefit more from the protective effects of social relationships because they are more exposed to, or vulnerable to, stressors such as health declines and other life events associated with aging (Chan, Anstey, Windsor, & Luszcz, 2011). There is longitudinal evidence that social support is related to depressive symptoms among older people. In particular, received instrumental and emotional support and perceived social support (i.e., positive evaluation of support) from family and friends have been associated with fewer depressive symptoms at follow-up in the general population of older adults (Russell & Cutrona, 1991; Sonnenberg et al., 2013). Although most evidence is from Western cultures, there is some evidence for a protective effect of support in other cultures (Chao, 2011, 2014; Zimmer & Chen, 2012).

However, it is increasingly realized that negative aspects of social relationships should be considered alongside positive social support. Positive relationship quality is indicated by mutual understanding and openness. Negative aspects of the relationship include criticism and negative feelings, such as feeling let down or feeling that the other person is making too many demands. Conflict and criticism may coexist with emotional and instrumental support (Bradbury & Karney, 2004). There is evidence that negative aspects of social relationships are related to depressive symptoms in later life independently of positive support (Ingersoll-Dayton, Morgan, & Antonucci, 1997; Newsom, Nishishiba, Morgan, & Rook, 2003; Newsom, Rook, Nishishiba, Sorkin, & Mahan, 2005).

As noted, the association between support and depressive symptoms may be bidirectional over time. Studies also show that depressive symptoms are prospectively associated with poorer perceived social support at follow-up (Gurung, Taylor, & Seeman, 2003; Leskela et al., 2008) and with greater negative interactions at follow-up (Krause & Rook, 2003). Recovery from depression leads to improved evaluation of one’s social support, especially in older compared with younger adults (Patten, Williams, Lavorato, & Bulloch, 2010). Despite this suggestive evidence, there has, so far, been little empirical estimation of the bidirectional nature of the associations between trajectories of positive and negative social support and trajectories of depressive symptoms. The main aim of this study was to address this gap using longitudinal data from a nationally representative cohort study set in England.

Social support may be derived from many different relationships including the spouse or partner (hereon referred to simply as spouse), other close family, friends, and others (Fiori, Antonucci, & Cortina, 2006). The current study focuses on spousal social support for two main reasons. Spouses and children make up a greater portion of the social network of older compared with younger adults (Aartsen, van Tilburg, Smits, & Knipscheer, 2004), and the spouse is the most frequently nominated close person in older age (Antonucci, Akiyama, & Takahashi, 2004; Cornwell, Laumann, & Schumm, 2008; Cornwell, Schumm, Laumann, & Graber, 2009). There is evidence that a good relationship with the spouse is more strongly related to lower risk of depressive symptoms than other social relationships in later life (Teo, Choi, & Valenstein, 2013). The quality of the spousal relationship is, therefore, important in older age. In line with the functional-specificity model (Weiss, 1974), the various forms of support provided within the spousal relationship may have a different impact when provided by another relationship. For example, the existence of a spousal relationship (and a relationship with children) has been related to sense of attachment among older people, whereas the existence of other types of relationship was not related to sense of attachment (Simons, 1984). However, reassurance of self-worth from those outside the family has been more strongly linked to positive affect than that derived from those within the family (Felton & Berry, 1992). Whereas low social support among unmarried people may indicate that they have few social connections, among married people, it may indicate an unhappy marriage or difficulty in handling disagreements within the marriage, and it may in itself be a source of stress (Coyne & DeLongis, 1986). Therefore, rather than consider social support from multiple sources combined (Chou & Chi, 2003; Krause, Liang, & Yatomi, 1989; M. G. Taylor & Lynch, 2004), we limit our investigation to change in support provided within the spousal relationship and its association with changes in depressive symptoms. This focus should be noted when interpreting the findings, however, because the magnitude of the association between spousal support and depressive symptoms may be modified by the presence of, and quality of, relationships with others who make up the closest part of the social convoy (Birditt & Antonucci, 2007).

In addition, changes in spousal support in older age may show a different pattern than changes in social support across multiple sources. Generally, studies indicate rising (Cornman, Lynch, Goldman, Weinstein, & Lin, 2004; Cornwell et al., 2009; Martire, Schultz, Mittlemark, & Newsom, 1999; Shaw, Krause, Liang, & Bennett, 2007) or stable (M. G. Taylor & Lynch, 2004; van Tilburg, 1998) levels of emotional support and declining negative interactions (Fuller-Iglesias, Webster, & Antonucci, 2015; Shaw et al., 2007) with age among all sources combined (in line with socioemotional selectivity theory; Carstensen, Isaacowitz, & Charles, 1999), but not with the spouse in particular. Mixed positive and negative feelings are particularly high for the spousal relationship (Fingerman, Hay, & Birditt, 2004), and longitudinal studies point to declining marital quality (Birditt, Jackey, & Antonucci, 2009; Gurung et al., 2003; Umberson, Williams, Powers, Liu, & Needham, 2006) and declining satisfaction with marital support (VanLaningham, Johnson, & Amato, 2001) over time. This may be because the possibility to withdraw from an unsupportive relationship is more difficult in the case of the relationship with the spouse compared with other relationships. It is not yet clear whether positive and negative support change with age in the same way for older men and women. Longitudinal studies are mixed in finding not only that changes are not modified by gender (VanLaningham et al., 2001) but also that gender differences in poor marital quality are most marked in older age (Umberson & Williams, 2005), and that women experience greater increases in negative interactions with their spouses than men (Gurung et al., 2003).

Previous longitudinal examinations of the association between spousal support and depressive symptoms were based on two waves of data. One did not consider bidirectionality (Stafford, McMunn, Zaninotto, & Nazroo, 2012). One showed baseline marital discord predicted depressive symptoms at 2-year follow-up and baseline depression predicted subsequent marital discord among middle-aged and older adults (Whisman & Uebelacker, 2009). The only other study to consider bidirectional associations between spousal support and depression was based on younger twins (less than 58 years). That study showed that female twins with greater spousal support had lower risk of major depression 4 years later, and that baseline major depression was associated with greater problems with spouse at follow-up. These longitudinal associations were not fully explained by shared genetic factors (Wade & Kendler, 2000).

In longitudinal studies that captured social support from multiple close persons (not only the spouse), one found a strong negative association between baseline satisfaction with support and depressive symptoms at 18-month follow-up but no association between baseline depressive symptoms and later satisfaction with support (Krause et al., 1989). However, another found baseline depressive symptoms were positively associated with support from a household member 3 years later (Chou & Chi, 2003). We identified only one existing study that modeled depressive symptoms and social support trajectories. Based on multiple data points over a 10-year period, increases in perceived social support summed across all sources were found to be correlated with decreases in depressive symptoms (M. G. Taylor & Lynch, 2004).

In summary, although there is some evidence for a bidirectional association, trajectories of spousal support and depressive symptoms in later life as related functions do not appear to have been described. We extend existing work to include multiple waves to better estimate cross-sectional and longitudinal bidirectional associations, considering changes in depressive symptoms and social support as processes that may be related to each other in older age using bivariate growth models. We also consider positive and negative aspects of the spousal relationship separately. Available data led us to focus on perceived support. Measures of perceived support have the advantage of allowing for the fact that not all partner’s attempts at support are viewed as helpful, and they correlate well with reported marital quality. However, they do not necessarily capture the number and quality of supportive interactions or behaviors from the spouse (Dehle, Larsen, & Landers, 2001).

The current study aimed to test associations between changes in positive and negative spousal support and changes in depressive symptoms captured on up to five occasions from 8 years of follow-up in a nationally representative sample of older men and women living in England. We hypothesized a bidirectional association between depressive symptoms and support such that (a) baseline higher positive support would be associated with a stable or declining trajectory of depressive symptoms and baseline negative support with an increasing trajectory of depressive symptoms and that (b) a higher number of depressive symptoms at baseline would be associated with a decline in positive support (or an increase in negative support) over time. We also considered gender differences in trajectories and in the association between spousal support and depressive symptoms, given previous evidence that women’s mental health may be more negatively affected by low support (Kendler, Myers, & Prescott, 2005).

## Data and Method

The data for this study came from the English Longitudinal Study of Ageing (ELSA). ELSA is a sample of people aged 50 and older living in England. It was drawn from households that responded to the Health Survey for England (HSE). Individuals were classified as core ELSA sample members at Wave 1 if they were included in the HSE and were aged 50 in 2002, when the first wave of ELSA took place. A total of 11,391, 67% of eligible sample members, took part in Wave 1 of ELSA. More detail on the sampling and response rates for ELSA is given elsewhere (R. Taylor et al., 2007). Every 2 years, data are collected by a trained interviewer in the participant’s home and, after a computer-assisted personal interview, respondents are also asked to fill in a self-completion questionnaire. Data from core members from the first five waves were used here and were accessed through the U.K. Data Service (UK Data Service, 2017). Ethical clearance for ELSA was obtained from the Multicentre Research and Ethics Committee.

### Measures

An eight-item version of the Center for Epidemiologic Studies Depression Scale (CES-D) was used to capture depressive symptoms by interview. This tool has been validated against the full CES-D (Turvey, Wallace, & Herzog, 1999) and predicts mortality in community samples (Turvey, Schultz, Beglinger, & Klein, 2009). In the self-completion questionnaire, participants were asked whether they had experienced each symptom much of the time during the past week (possible responses “yes” or “no”). Positive support from the spouse or partner was captured in the self-completion questionnaire by three items covering empathy, dependability, and confiding. The item wordings were as follows: “How much do they really understand the way you feel about things?” “How much can you rely on them if you have a serious problem?” and “How much can you open up to them if you need to talk about your worries?” Possible responses ranged from *a lot* (coded 3) to *not at all* (coded 0) and were summed to create a positive support from spouse scale (ranging from 0 to 9; Cronbach’s α = .79). Negative support was captured by three items covering criticism, being let down, and annoyance, and was coded in the same way (with high scores indicating high negative support; Cronbach’s α = .62). The items were, “How much do they criticize you?” “How much do they let you down when you are counting on them?” and “How much do they get on your nerves?”

Age (from 50 to 99 years), gender (male, female), and educational attainment (secondary and above, primary and below) were identified as potentially important covariates and measured at baseline interview. At each wave, respondents were coded as having a limiting long-standing illness if they stated they had a long-standing illness, disability, or infirmity, *and* that this limited their activities in any way. Household wealth quintile (including housing and nonhousing wealth minus debts but excluding pension wealth) was also captured at each wave.

### Statistical Method

Longitudinal changes in depressive symptoms and spousal support were first described separately. The number of depressive symptoms was analyzed as a continuous variable. Using up to five waves of data, baseline (intercept) and within-person linear change (slope, per 2-year increase in follow-up time) in depressive symptoms were estimated using a latent growth model. Latent intercept and slope were controlled for gender and age (centered at *M* = 63 years). Levels of positive and negative support were also analyzed as continuous variables, and baseline and within-person change in these variables were estimated in the same way in separate univariate models. We present aging vector graphs of predicted scores (depressive symptoms, positive support, and negative support) to show visually the level of each score at baseline, and the direction and amount of change throughout the age range of our sample. Each arrow represents the predicted origin and change in each score for an 8-year birth cohort. The graphs reveal both trends by age of the sample at baseline *and* cohort-specific within-person changes over time in depressive symptoms, positive support, and negative support.

**Figure 1.**
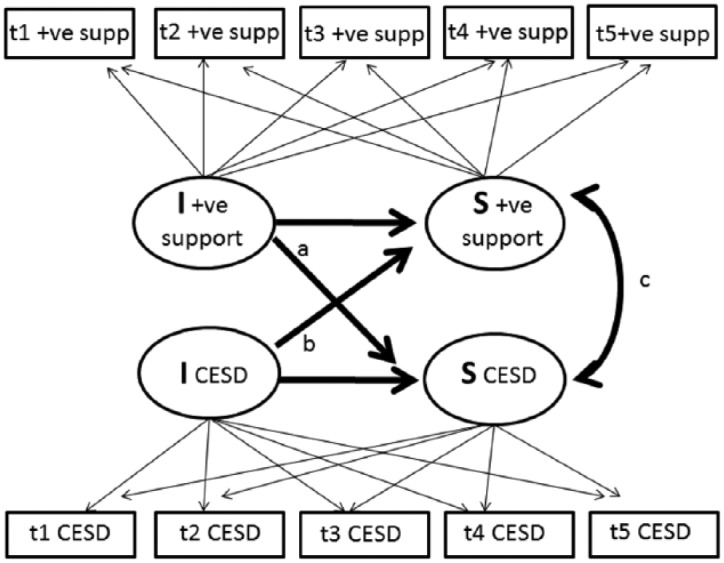
Simplified schematic of parallel process model describing associations of change in positive support with change in CES-D depressive symptoms.

The associations between the latent intercepts and latent slopes of depressive symptoms and positive support were then estimated using a two-parallel process latent growth model (Figure 1; Muthén, 1998-2010). Coefficient *a* represents the association between positive support at baseline (I +ve support) and change in depressive symptoms (S CES-D). Coefficient *b* represents the association between baseline depressive symptoms (I CES-D) and change in positive support (S +ve support). Coefficient *c* represents the correlation between slopes, with a negative and statistically significant correlation indicating that increases in positive support are associated with decreases in depressive symptoms. This approach was repeated to estimate associations between depressive symptoms and negative support. Baseline age and education and time-varying wealth and limiting long-term illness were controlled, and each of these variables was grand mean centered. We show estimates for men and women combined (with adjustment for gender), and also, based on multiple group analysis, with gender as the grouping variable. In sensitivity analysis, we tested whether associations between depressive symptoms and spousal support depended on age (i.e., we included age [coded as 50-64 and 65+ years] as a grouping variable). Coefficients *a, b*, and *c* did not statistically significantly differ by age group (data available from authors). We note the limited age range in these data and do not interpret this finding as indicating that the association between spousal support and depressive symptoms would be constant across all age adults (see Patten et al., 2010).

We evaluated several goodness-of-fit indices to determine the fit of the models. Chi-square is sensitive to sample size; in large samples, it will tend to be statistically significant when there are only minor misspecifications of the model (Hu & Bentler, 1999). Therefore, we employed the comparative fit index (CFI) and the root mean square error of approximation (RMSEA). The CFI is based on the chi-square statistic; it ranges between 0 and 1, with values close to 1 indicating a more acceptable fit to the data. The RMSEA represents closeness of fit, with values below .05 representing close fit of the model, although others suggest a value of .06 (Hu & Bentler, 1999). To handle missing covariate data, we used full information maximum likelihood estimation, which computes parameter estimates on the basis of all available data under the assumption that data are missing at random (Enders & Bandalos, 2001). Ethnicity (coded as White or non-White) and government office region were included as auxiliary variables because these, along with health and socioeconomic characteristics, are associated with missingness (R. Taylor et al., 2007).

Analyses were run using MPlus version 7.11 software.

### Analytical Sample

Wave 1 sample members who had a spouse were included in the analysis. Of the 11,391 who took part in Wave 1, 1,213 did not answer the self-completion questionnaire. Of the remaining 10,178 participants, 2,893 did not have a spouse. After exclusion of those with missing depressive symptoms or spousal support data, the current study was based on 7,171 participants. Of these, 3,639 provided data at all five waves (3,412 on depressive symptoms, 2,497 on positive support, and 2,487 on negative support), 1,058 at three or four waves, and 1,934 at one or two waves.

## Results

Participants in the current study had a mean age of 62.9 years and just more than 30% had a limiting long-term illness (Table 1). Sample members who were not included in the current study were older, less highly educated, and less wealthy than those who were included. Those who provided data from fewer waves also had higher mean age, had lower levels of educational attainment, were less wealthy, had lower levels of positive support and higher levels of negative support, and had higher mean depressive symptoms than those with data at all five waves.

**Table 1.** Baseline Characteristics of *N* = 7,171 Study Participants.

	*M* (*SD*)	Analytical sample *n*
Age	62.9 (8.9)	7,171
Positive support	7.9 (1.6)	7,171
Negative support	2.4 (1.8)	7,112
Number of depressive symptoms	1.3 (1.7)	7,078
	%	Analytical sample *n*
Female	48.6	3,487
Household wealth quintiles		
Richest	25.8	1,811
Fourth	23.4	1,640
Third	21.2	1,491
Second	18.5	1,296
Poorest	11.2	786
Low education	51.0	3,656
White	97.7	7,007
Limiting long-term illness	31.3	2,246
Government office regions		
North East	6.3	448
North West	12.7	910
Yorkshire and The Humber	11.5	826
East Midlands	9.9	708
West Midlands	10.6	763
East of England	12.3	879
London	8.2	585
South East	16.9	1,209
South West	11.8	843

### Univariate Trajectories

Univariate changes in depressive symptoms and in support over time, controlling for gender and baseline age, were examined (Table 2 and Supplementary Figure 1). Compared with men, women had, on average, 0.39 more depressive symptoms at baseline. The mean number of symptoms increased over time at a rate of 0.019 symptoms per 2 years, and there was no evidence that this increase differed for men and women. From age 63 onward (the centering point for the age variable), higher age was positively related to baseline depressive symptoms and positively related to increasing depressive symptoms over time. These data indicate a decrease in depressive symptoms over 8 years of follow-up in the youngest cohort and a nadir for depressive symptoms in the midsixties. The intercept and slope variance estimates indicate significant variation between individuals in both the initial level of depressive symptoms and change in symptoms over time.

**Table 2.** Univariate Growth Curve Models for Changes Over Time in Mean (a) Number of Depressive Symptoms, (b) Positive Spousal Support, and (c) Negative Spousal Support.

	Depressive symptoms	Positive support	Negative support
	Estimate (*SE*)	Estimate (*SE*)	Estimate (*SE*)
Growth factors			
Intercept	1.10 (0.03)	8.21 (0.03)	2.34 (0.03)
Intercept variance	1.57 (0.05)	1.74 (0.04)	1.90 (0.05)
Slope (per 2 years)	0.019 (0.009)	−0.036 (0.008)	0.050 (0.01)
Slope variance (per 2 years)	0.050 (0.005)	0.037 (0.004)	0.023 (0.005)
Associations between growth factors and covariates			
Intercept on female	0.39 (0.04)	−0.52 (0.04)	0.18 (0.04)
Intercept on age (centered at 63 years)	0.009 (0.002)	0.004 (0.002)^*ns*^	−0.006 (0.002)
Slope on female	0.015 (0.013)^*ns*^	−0.027 (0.012)	0.033 (0.012)
Slope on age (centered at 63 years)	0.006 (0.001)	−0.002 (0.001)	0.000 (0.001)^*ns*^
Model fit			
CFI	.987	.986	.996
RMSEA	.028	.056	.018
*N*	7,140	7,171	7,112

*Note. ns* = nonsignificant; CFI = comparative fit index; RMSEA = root mean square error of approximation.

Univariate changes in support from the spouse are also shown in Table 2. Positive support from the spouse was lower at baseline for women compared with men (by 0.52 points, around one third of a standard deviation in positive support; Supplementary Figure 2). On average, positive support decreased over time and decreased more for women than for men. Age was not related to positive support at baseline but was negatively associated with positive support slope, indicating that older participants experienced greater declines over time compared with younger participants. Negative support from the spouse was slightly higher at baseline for women and for younger cohorts (Table 2 and Supplementary Figure 3). There was an overall increase in negative support over time, particularly for women.

### Joint Trajectories of Positive Support and Depressive Symptoms

Table 3 summarizes the correlations between changes in positive support and depressive symptoms based on a two-parallel process model. In the model with men and women combined, adjusted for age, gender, wealth, education, limiting long-term illness, and depressive symptoms, there was no significant change in positive support over time. This is in contrast to the age- and gender-adjusted analysis presented in Table 2, in which positive support was found to decrease over time. Differences between these models indicate that time-varying socioeconomic circumstances and limiting long-term illness explained declines in positive support. The mean number of depressive symptoms, however, increased over time even after adjustment for these health and socioeconomic factors at a rate of 0.28 symptoms per 2 years. In line with the first hypothesis, those with higher baseline support had a smaller increase in number of depressive symptoms (standardized regression coefficient −0.26). Baseline depressive symptoms were negatively associated with change in positive support (standardized regression coefficient −0.21). In other words, in line with the second hypothesis, those with more baseline symptoms had a greater decrease in positive support. The magnitude of these coefficients was very similar; there was no suggestion that one direction dominated. Changes in the number of depressive symptoms and changes in positive support were negatively correlated. In other words, those who experienced declining positive support tended to experience an increase in number of depressive symptoms during the same period. Estimates from the multiple group model show that the same associations were seen when men and women were considered separately. That is, there was no evidence of gender differences in the associations between trajectories of positive spousal support and depressive symptoms.

**Table 3.** Two-Parallel Process Positive Support and Depression.

	All	Men	Women
	Estimate (*SE*)	Estimate (*SE*)	Estimate (*SE*)
Positive support growth factors			
Intercept	7.96 (0.02)	8.21 (0.02)	7.68 (0.03)
Intercept variance	1.67 (0.04)	0.92 (0.04)	2.36 (0.07)
Slope (per 2 years)	−0.01 (0.01)^*ns*^	−0.00 (0.01)^*ns*^	−0.01 (0.02)^*ns*^
Slope variance (per 2 years)	0.03 (0.003)	0.03 (0.003)	0.04 (0.005)
Depressive symptoms growth factors			
Intercept	1.29 (0.02)	1.11 (0.02)	1.49 (0.03)
Intercept variance	1.06 (0.03)	0.92 (0.04)	1.19 (0.05)
Slope (per 2 years)	0.28 (0.04)	0.30 (0.07)	0.26 (0.05)
Slope variance (per 2 years)	0.03 (0.004)	0.03 (0.004)	0.02 (0.006)
	Standardized estimate (*SE*)	Standardized estimate (*SE*)	Standardized estimate (*SE*)
Associations between growth factors			
Intercept depression on positive support slope	−0.21 (0.04)	−0.20 (0.06)	−0.21 (0.06)
Intercept positive support on depression slope	−0.26 (0.04)	−0.20 (0.05)	−0.31 (0.06)
Depression slope with positive support slope	−0.54 (0.08)	−0.62 (0.11)	−0.48 (0.14)
Model fit			
CFI	.931	.925	
RMSEA	.038	.039	
*N*	7,171	7,171	

*Note.* Adjusted for age, sex, education, ethnicity, government office region, wealth, and limiting long-standing illness, all mean centered. *ns* = nonsignificant; CFI = comparative fit index; RMSEA = root mean square error of approximation.

### Joint Trajectories of Negative Support and Depressive Symptoms, and Gender Differences

Table 4 summarizes changes in negative support and depressive symptoms and associations between the growth factors. In the model for men and women combined, there was no overall change in negative support in the fully adjusted model. More detailed analysis revealed that this was due to the adjustment for depressive symptoms at baseline (data available from the authors). There was an overall decline in number of depressive symptoms over time (of 0.10 symptoms per 2 years) in the fully adjusted model. In other words, adjustment for additional covariates explained the increases in depressive symptoms seen between Waves 1 and 5 in the age- and gender-adjusted univariate model. There was a positive association between baseline negative support and increasing depressive symptoms. There was also a positive association between baseline number of depressive symptoms and change in negative support; those with more symptoms at baseline experienced increasing negative support over time. There was no evidence that changes in negative support were correlated with changes in number of depressive symptoms. These associations provide support for the first and second hypotheses; however, there was evidence, albeit weak, of gender difference in some of these associations. The multiple group analysis indicated that estimates representing associations between the growth factors were similar for men and women, with the exception that the positive association between baseline depressive symptoms and increase in negative support was larger for men than women (*p* < .05). Gender differences in other estimates did not attain statistical significance though there was a suggestion that the positive association between baseline negative support and depressive symptoms slope was a little larger among women (β = .42) than men (β = .28) and that women (β = .05), but not men (β = −.02), experienced an increase in negative support through follow-up, conditional on all other covariates.

**Table 4.** Two-Parallel Process Negative Support and Depression.

	All	Men	Women
	Estimate (*SE*)	Estimate (*SE*)	Estimate (*SE*)
Negative support growth factors			
Intercept	2.43 (0.08)	2.34 (0.03)	2.53 (0.03)
Intercept variance	1.17 (0.04)	1.38 (0.05)	2.14 (0.07)
Slope (per 2 years)	0.01 (0.01)^*ns*^	−0.02 (0.01)	0.05 (0.02)
Slope variance (per 2 years)	0.01 (0.004)	0.01 (0.004)	0.02 (0.006)
Depressive symptoms growth factors			
Intercept	1.29 (0.02)	1.11 (0.02)	1.49 (0.03)
Intercept variance	1.06 (0.03)	0.92 (0.04)	1.19 (0.05)
Slope (per 2 years)	−0.10 (0.01)	−0.09 (0.02)	−0.10 (0.02)
Slope variance (per 2 years)	0.02 (0.004)	0.03 (0.004)	0.02 (0.006)
	Standardized estimate (*SE*)	Standardized estimate (*SE*)	Standardized estimate (*SE*)
Associations between growth factors			
Intercept depression on negative support slope	0.44 (0.04)	0.62 (0.13)	0.25 (0.11)^a^
Intercept negative support on depression slope	0.35 (0.04)	0.28 (0.13)	0.42 (0.08)
Depression slope with negative support slope	0.14 (0.17)	−0.01 (0.24)	0.34 (0.26)
Model fit			
CFI	.934	.931	
RMSEA	.033	.034	
*N*	7,171	7,171	

*Note.* Adjusted for age, sex, education, ethnicity, government office region, wealth, and limiting long-standing illness, *ns* = nonsignificant; CFI = comparative fit index; RMSEA = root mean square error of approximation.

a Intercept depression on negative support slope differed for men and women (*p* < .05).

## Discussion

Based on five waves of data spanning 8 years of follow-up, we found an average decrease in positive support and increase in negative support from spouses in age- and gender-adjusted models. These slopes were explained by circumstances including illness and wealth that also change in later life, and by levels of depressive symptoms at the start of follow-up, discussed later. Changes were larger in magnitude for women compared with men. This aligns with the small literature focused on marital quality, which has found declining satisfaction with support from the spouse and increasing negative spousal exchanges in later life (Gurung et al., 2003; VanLaningham et al., 2001). Depressive symptoms also increased through follow-up for those in their midsixties and above but declined or were stable for those who were younger at baseline. Our findings concur with several longitudinal studies, which show increases in depressive symptoms with age from the midsixties onward (Burns et al., 2013; Davey, Halverson, Zonderman, & Costa, 2004; Mirowsky & Kim, 2007; M. G. Taylor & Lynch, 2004), partly due to declines in physical health and proximity to death (Sutin et al., 2013), although others have identified groups with differing trajectories (Melchior et al., 2013). Assessment of the age-related change in depressive symptoms was not the main aim of our study, but we note that the changes in depressive symptoms seen here were small in magnitude.

The main focus of our study was to examine the evidence for bidirectional associations of depressive symptoms and spousal support. We built on previous studies, which have tended to focus on one as the exposure and the other as the outcome of interest. As hypothesized, higher baseline positive support was associated with greater decrease in depressive symptoms over time, controlling for age, gender, education, wealth, and limiting long-term illness. Similarly, baseline depressive symptoms were negatively associated with trajectories of positive support. The associations between baseline positive support and changes in depressive symptoms were the same magnitude as those between baseline depressive symptoms and changes in positive support. We also found correlations between changes in positive support and changes in depressive symptoms. That is, given initial levels of positive support from the spouse, new information of relevance for understanding changes in depressive symptoms is gained by knowing about *changes* in that support.

The same conclusion cannot be drawn for the associations between trajectories of negative support and depressive symptoms, however, because there was a suggestion that these may depend on gender. In particular, there was evidence that baseline depressive symptoms were more strongly associated with a subsequent increase in negative support among men than women. Based on the estimated coefficients, there was a tendency toward a steeper association between baseline negative support and increasing depressive symptoms over time among women compared with men, though the gender difference did not attain statistical significance. Put together, these findings could suggest that the directionality of the association between depressive symptoms and negative support depends on gender. This warrants testing in other studies, given that statistical evidence was not strong and that we did not have a priori reasons to expect this result. Gender differences have been found in one previous study, in younger adults (aged 21-58 years; Kendler et al., 2005), which showed that positive support showed a stronger protective association for major depression among women. That study did not consider positive and negative aspects of support separately.

Bidirectional causal associations are conceptually plausible. Spousal support may reduce depressive symptoms by facilitating access to resources that buffer the potentially detrimental effects of stressors (Berkman, Glass, Brissette, & Seeman, 2000). Supportive spouses may also motivate self-care and promote a sense of belonging, which reduces the likelihood of depressive symptoms (Krause, 2007). Spousal support may also lead to lower marital conflict, which is, in turn, associated with lower risk of depressive symptoms (Cramer & Jowett, 2010). Depressive symptoms and depressive cognition, however, can reduce a person’s ability to recognize and use the positive support being offered as well as increasing the sensitivity to negative aspects of social relationships (Alloy et al., 1999; Maher, Mora, & Leventhal, 2006).

### Strengths and Limitations

These findings are based on data from a large, representative sample of people aged 50 and above living in England, though may not be generalizable to other cultures. The analytical method allowed for heterogeneity, that is, differences in spousal support and depressive symptom trajectories among older adults (Krause, 1999). Some limitations must be considered. The internal consistency of the negative support scale was rather low, and this may result in some underestimation of the association between negative support and depressive symptoms. Furthermore, both positive and negative aspects of support were captured by only three items and did not represent the multiple dimensions of support that may be relevant for depressive symptomatology (Finch, Okun, Pool, & Ruehlman, 1999). The study is based on self-reported data. These findings do provide stronger evidence than that provided by cross-sectional studies, however, because the associations between initial levels in one process and changes in the other process are not driven by cross-sectional correlations.

As with all longitudinal studies, loss to follow-up may have introduced bias. Depressive symptoms, poor own and spousal health, and widowhood are associated with dropout. This may result in an underestimation of the real increase in negative support and decrease in positive support in the general population because poor health may place a strain on the marital relationship. In addition, we expect that our analysis somewhat underestimated the true increases in depressive symptoms and the strength of the association between depressive symptoms and both negative and positive support. To minimize the potential bias derived from missing data due to loss to follow-up, we used full information maximum likelihood estimation. Thus, the partial trajectories of those who did not provide data at later waves were able to be estimated.

There are a number of variables that we have not been able to consider in this analysis. The possibility of confounding by unmeasured factors (e.g., neuroticism or another element of personality disposition) cannot be ruled out (Lewis, Bates, Posthuma, & Polderman, 2014). Individuals, rather than dyads, were the unit of analysis. We measured perceived support from an individual’s perspective but did not have any information capturing interactions within the partnership that may be most useful for developing interventions (Coyne & DeLongis, 1986). Although we captured negative aspects of the spousal relationship, we did not explicitly capture spousal conflict. Previous studies show that couple’s management of conflict is linked to depressive symptoms (Du Rocher Schudlich, Papp, & Cummings, 2011). Furthermore, we considered negative and positive aspects of the spousal relationship separately and did not explore their joint effects on depressive symptoms, though previous evidence indicates that they influence each other over time (Bradbury & Karney, 2004) and that they may modify each other’s effects (DeLongis, Capreol, Holtzman, O’Brien, & Campbell, 2004). Our study focused on the spousal relationship and we did not investigate changes in positive and negative support in other relationships, or possible modification of the link between spousal support and depressive symptoms according to support derived from other sources (Birditt & Antonucci, 2007). Cohabiting partnerships were also included in our analysis, though we did not distinguish between married and cohabiting couples.

### Implications for Research and Practice

The finding of a bidirectional association between depressive symptoms and support from the spouse or partner has two key implications. First, initiatives to reduce depressive symptoms may have consequences for the spousal relationship. Second, the context of the spousal relationship is relevant to consider in treatment for depression among married and cohabiting older people. This observational, epidemiological study is one step in understanding these associations. Conceptually, spousal relationship quality might be expected to improve with age due to the increasing emotional control and emotional understanding, and greater familiarity that comes with experience (Carstensen, Gottman, & Levenson, 1995; Charles, 2010; Charles & Piazza, 2007). However, chronic stressors and vulnerabilities that might negatively affect the quality of the spousal relationship tend to increase with age (Charles, 2010). A previous study showed negative interactions with the spouse or partner were highest among those aged 75+ compared with younger ages (Akiyama, Antonucci, Takahashi, & Langfahl, 2003). We also found declines in spousal relationship quality with advancing age in models, which were adjusted for age and gender, and our results shed some light on factors that might underlie this. In the fully adjusted models, which were additionally controlled for baseline depressive symptoms and time-varying illness and wealth, there was no evidence of significant change in positive or negative support from the spouse. The quality of support derived from the relationship with the spouse suffers in the presence of depressive symptoms, limiting illness and low wealth. This highlights a potential intervention point for the improvement of the quality of the spousal relationship in older age. In addition to the known societal and individual burden of depressive symptoms, this study emphasizes the potential cost to a couple’s relationship quality.

Previous studies note the concordance in depressive symptoms among spouses, due to assortative mating, social homogamy, and shared environment (Pradeep & Sutin, 2015). Our study finds that the quality of spousal relationship is one element of the shared environment that is relevant for depressive symptoms. Indeed, other evidence suggests that the spousal relationship, to a greater extent than other relationships, is a key determinant of depressive symptoms (Teo et al., 2013). We did not have data reported by the spouse but future research might investigate the extent to which any correlation between trajectories of depressive symptoms of spouses is due to positive and negative support. The presence of depressive symptoms might limit the support that spouses can give each other. Initiatives to improve the quality of a couple’s relationship, both by facilitating positive forms of support and reducing negative aspects, may be one approach to reducing depressive symptoms among older people. This may include interventions provided to couples as well as to individuals facing challenges associated with aging.

## Supplementary Material

Supplementary material
