# Supplementary figures and images for: Joint Trajectories of Spousal Social Support and Depressive Symptoms in Older Age
Source: J Aging Health. 2017 Dec 14;31(5):760–82. doi: 10.1177/0898264317747077 (PMC6495403; doi:10.1177/0898264317747077)

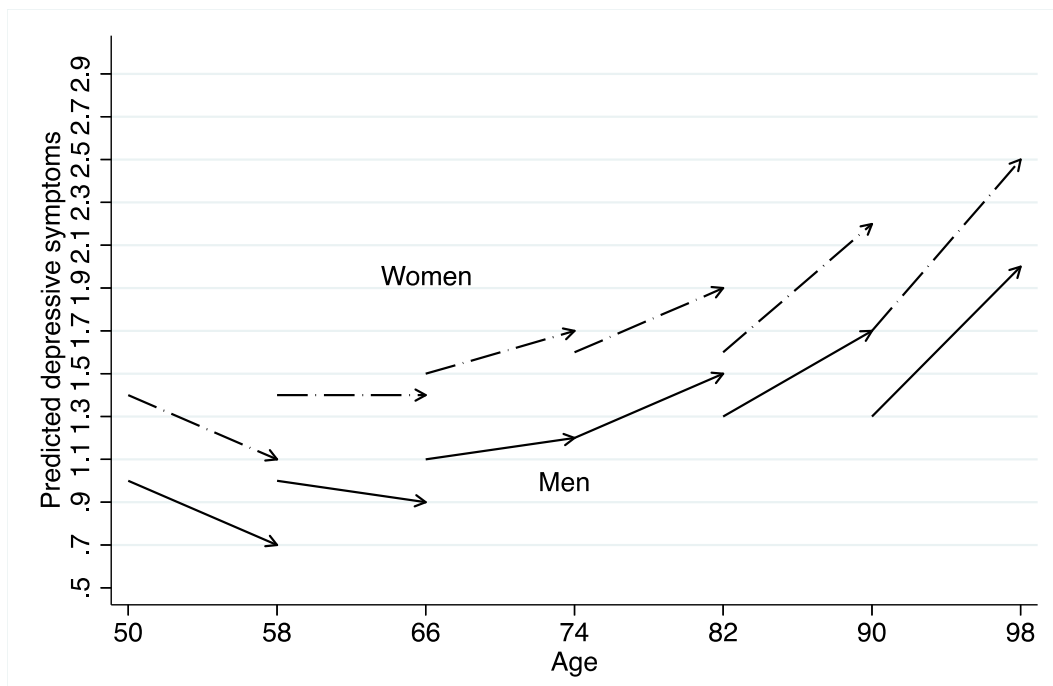

Figure 1

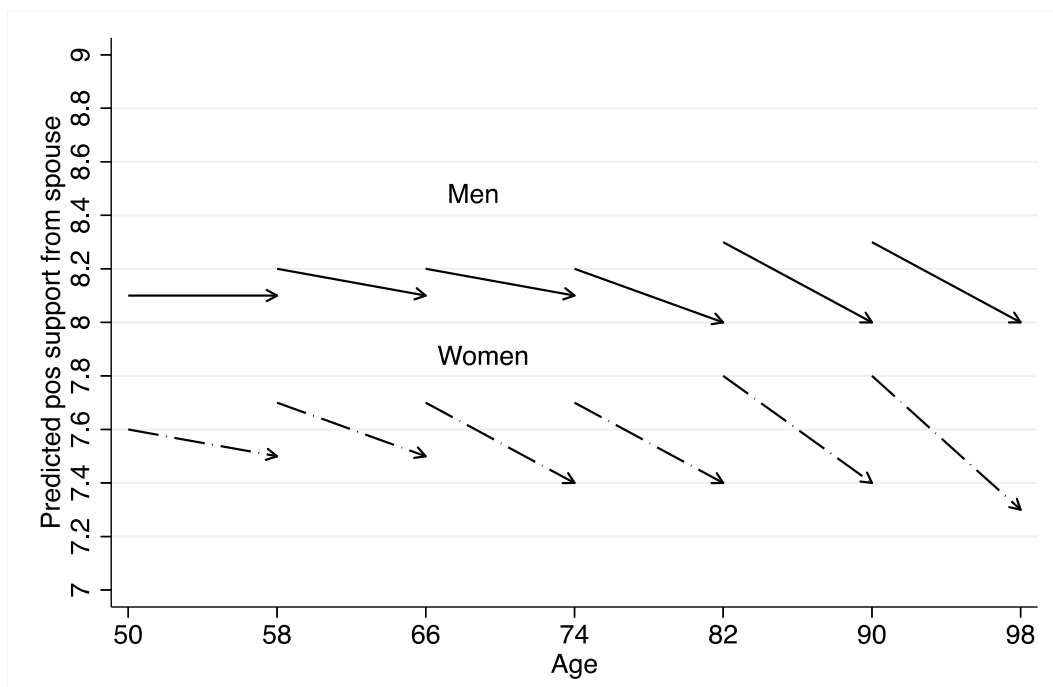

Figure 2

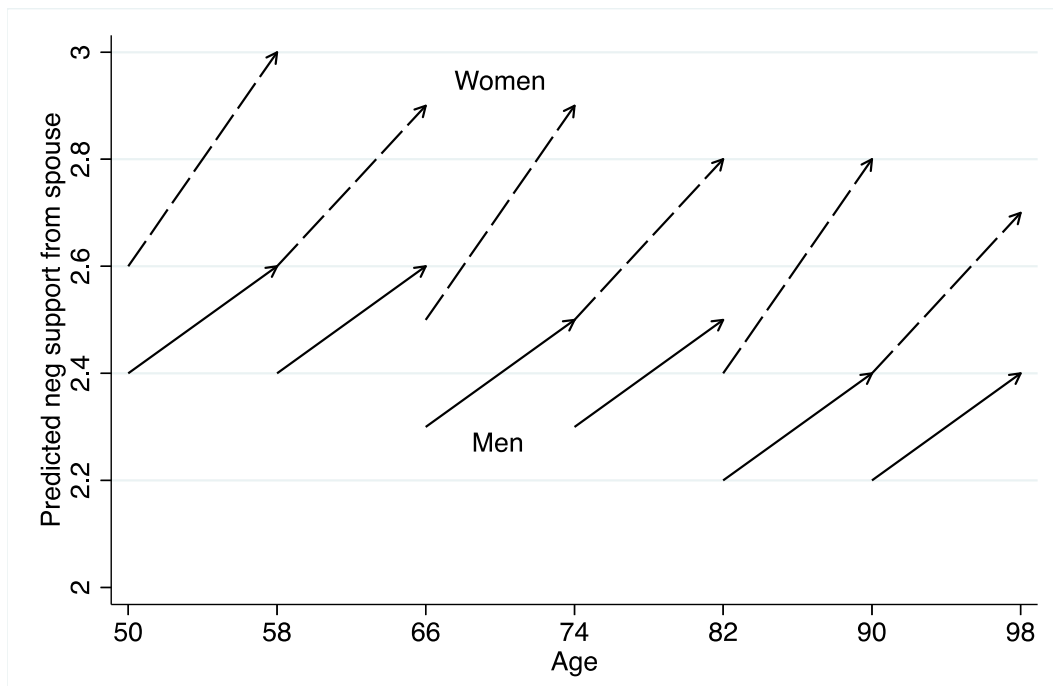

Figure 3

Supplement: Supplementary material [file Supplementary_Figures.pdf]
